# Class IIa histone deacetylase inhibition ameliorates acute kidney injury by suppressing renal tubular cell apoptosis and enhancing autophagy and proliferation

**DOI:** 10.3389/fphar.2022.946192

**Published:** 2022-07-22

**Authors:** Jialu Li, Chao Yu, Fengchen Shen, Binbin Cui, Na Liu, Shougang Zhuang

**Affiliations:** ^1^ Department of Nephrology, Shanghai East Hospital, Tongji University School of Medicine, Shanghai, China; ^2^ Department of Medicine, Rhode Island Hospital and Alpert Medical School, Brown University, Providence, RI, United States

**Keywords:** acute kidney injury, class IIa histone deacetylases, folic acid, ischemia/reperfusion, TMP269, apoptosis, autophagy, renal tubular cells

## Abstract

Expression and function of histone deacetylases (HDACs) vary with cell types and pathological conditions. Our recent studies showed that pharmacological targeting class IIa HDACs attenuated renal fibrosis, but the effect of class IIa HDAC inhibition on acute kidney injury (AKI) remains unknown. In this study, we found that four class IIa HDACs (4, 5, 7, 9) were highly expressed in the kidney of folic acid (FA) and ischemia/reperfusion (I/R)-induced AKI in mice. Administration of TMP269, a potent and selective class IIa HDAC inhibitor, improved renal function and reduced tubular cell injury and apoptosis, with concomitant suppression of HDAC4 and elevation of acetyl-histone H3. Mechanistical studies showed that TMP269 treatment inhibited FA and I/R-induced caspase-3 cleavage, Bax expression and p53 phosphorylation. Conversely, TMP269 administration preserved expression of E-cadherin, BMP7, Klotho and Bcl-2 in injured kidneys. Moreover, TMP269 was effective in promoting cellular autophagy as indicated by increased expression of Atg7, beclin-1, and LC3II, and promoted renal tubular cell proliferation as shown by increased number of proliferating cell nuclear antigen-positive cells and expression of cyclin E. Finally, blocking class IIa HDACs inhibited FA-and I/R-induced phosphorylation of extracellular signal-regulated kinases 1 and 2, and p38, two signaling pathways associated with the pathogenesis of AKI. Collectively, these results suggest that pharmacological inhibition of class IIa HDACs protects against AKI through ameliorating apoptosis, enhancing autophagy and promoting proliferation of renal tubular cells by targeting multiple signaling pathways.

## Introduction

Acute kidney injury (AKI) is a leading cause of death worldwide. It can be induced by diverse etiologies, including nephrotoxic drugs, ischemia/reperfusion injury, and sepsis (Venkatachalam et al., 2015; Kellum et al., 2021). Following AKI, kidneys can be fully recovered and resume their functions through repair and regeneration processes in most cases; however, severe AKI frequently results in maladaptive repair and eventually lead to irreversible renal damage and dysfunction, a situation necessitates renal replacement therapies (Venkatachalam et al., 2015; Agarwal et al., 2016). To date, there are no available medications that can effectively prevent or treat AKI. Thus, it is urgent to develop novel therapeutic approaches for AKI.

An effective treatment on AKI should be targeted on the key pathological processes of AKI. The development of AKI involves both early phase of renal tissue damage and late phase of renal regeneration. Injury to the kidney causes death of some renal tubular cells whereas uninjured and sublethal cells undergo repair and regeneration (Agarwal et al., 2016). During the process of regeneration, the remaining tubular epithelial cells dedifferentiate and proliferate to replace lost cells (Agarwal et al., 2016). Given that tubular cell death and regeneration are two major processes of AKI, an interference that targeting these two processes must be beneficial to renal structural and functional recovery. Recently, our and other studies have shown that some histone deacetylases (HDACs) are implicated in the pathogenesis of AKI in animal models (Shi et al., 2017; Tang et al., 2018; Hyndman, 2020). Thus, HDACs could be the potential targets for the treatment of AKI.

HDACs are a group of enzymes that induce global changes in gene transcription and protein expression through removal of acetyl groups from histone and non-histone proteins (Falkenberg and Johnstone, 2014; Zhao et al., 2018). Based on the structural homology analysis of acetylase in yeast germline, HDACs are divided into four categories. Class I HDACs include HDAC1, 2, 3, 8 and are widely expressed in tissues; Class II HDACs include HDAC4, 5, 6, 7, 9, 10, and are specifically expressed in tissues; class III HDACs are also called Sirtuin includes Sirtuin 1–7. Class IV HDAC only contains HDAC11. Class II HDACs are further divided into class IIa HDACs (HDAC4, 5, 7, 9) and class IIb HDACs (HDAC6, 10) according to the different domains (Ruijter et al., 2003). Numerous studies have revealed the implication of HDACs in the pathogenesis of chronic kidney diseases, such as diabetic nephropathy, polycystic kidney disease, and renal fibrosis (Liu and Zhuang, 2015; Liu, 2021). In general, activation of class I/II HDACs contributes to chronic renal diseases, whereas expression and activation of Sirtuins protects against acute and chronic kidney injury (Liu, 2021; Zhou et al., 2021).

In the past decade, great efforts have also been made to determine the role of HDACs in AKI. It has been reported that treatment with trichostatin A (TSA), a I/II HDAC inhibitor, significantly attenuated renal damage and improved renal function in a murine model of cisplatin-induced AKI (Liu et al., 2018); administration of valproic acid, another class I/II HDAC inhibitor, also promoted renal recovery in a murine model of AKI induced by ischemia/reperfusion (Costalonga et al., 2016). In contrast, application of MS-275, a selective class I HDAC inhibitor, led to aggravated renal injury and worsen renal dysfunction in murine model of ischemia/reperfusion-induced AKI (Liu et al., 2018). These data suggest that TSA and valproic acid-elicited renoprotection may be through inhibition of class II rather than class I HDACs. Since class II HDACs are composed of several isoforms and divided into class IIa HDACs and class IIb HDACs, it is necessary to use class or isoform-selective inhibitors to define their roles in AKI. Recently, by using tubastatin A, a selective inhibitor of HDAC6, we demonstrated that HDAC6 inhibition can protect the kidney from cisplatin- or rhabdomyolysis-induced AKI (Shi et al., 2017; Tang et al., 2018), suggesting that this isoform of class IIb HDACs is involved in the pathogenesis of AKI. Although our recent studies also revealed that pharmacologically targeting class IIa HDACs attenuated renal fibrogenesis, the effect of class IIa HDAC inhibition on nephrotoxic and ischemic AKI remains unexplored.

In this study, we assessed the therapeutic effect of TMP269, a highly selective class IIa HDAC inhibitor, on AKI and the mechanisms involved in two murine models-the first induced by folic acid and the second by ischemia/reperfusion. Our results indicated that inhibition of class IIa HDACs effectively protected the kidney from injury by suppressing apoptosis, enhancing autophagy and promoting renal tubular cell proliferation.

## Materials and methods

Antibodies to HDAC4, HDAC5, HDAC7, and HDAC9 were purchased from Santa Cruz Biotechnology (Dallas, TX, United States). Antibodies to NGAL, BMP7, and LC3 were purchased from Abcam Inc (Cambridge, MA United States). Antibodies to p-p53, p53, caspase-3, bcl-2, Bax, PCNA, P-ERK1/2, ERK1/2, E-Cadherin, and β-Actin were purchased from Cell Signaling Technology (Danvers, MA, United States). Antibodies to Atg7 and Beclin-1 were purchased from Arigo Institute (Taiwan, China). Antibodies to GAPDH and α-tubulin were purchased from Proteintech Group Institute (Wuhan, China). Secondary antibodies were purchased from LI-COR Biosciences (Lincoln, NE, United States). Serum creatinine (Scr) reagent kits and Blood urea nitrogen (BUN) reagent kits were purchased from Nanjing Jiancheng Bioengineering Institute (Nanjing, China). All other chemicals were purchased from Sigma (St. Louis, Missouri, United States).

### Models of Acute Kidney Injury and TMP269 treatment

Eight weeks-old C57/BL male mice (Shanghai SLAC Laboratory Animal Co., Ltd., China) weighing 20–25 g and murine models of AKI induced by either folic acid or I/R were used for the study. To establish FA-induced AKI, a single dose of FA (dissolved in 0.3 M NaHCO_3_) at 200 mg/kg was intraperitoneally administered. Control mice were injected with an equal volume of 0.3 M NaHCO_3_. To establish I/R-induce AKI, bilateral renal pedicels were clamped for 35 min to induce ischemia under anesthetization using the cocktail of ketamine (75 mg/kg, ip) and dexdomitor (0.5 mg/kg, ip), followed by reperfusion for 48 h. To examine the effect of class IIa HDAC inhibition on AKI, TMP269 at 50 mg/kg was intraperitoneally administered immediately after FA injection or reperfusion, and then given at the same dosage every 24 h for 48 h. TMP269 was dissolved in DMSO and DMSO alone treated mice were used as control. At least six mice were used in each group. At the end of experiments, animals were sacrificed by injecting an overdose of phenobarbital (100 mg/kg) and kidneys and blood were collected. All the animal studies were conducted in Tongji University (Shanghai, China) and the animal protocol was reviewed and approved by the Institutional Animal Care and Use Committee at Tongji University.

### Measurement of serum creatinine and blood urea nitrogen

Serum creatinine and blood urea nitrogen were determined by using automatic biochemistry assay (P800; Modular) according to the manufacture’s instruction.

### HE staining

The kidney tissue samples were dehydrated in 4% paraformaldehyde and then paraffin-embedded for sectioning. After dewaxing, renal tissue slices were stained with hematoxylin eosin. The tissue slices were photographed with an upright microscope, and 10 high-power fields (200 ×). Tubular injury was scored on a scale from 0 to 4: where 0 = normal tissue without injury; 1 = renal tubular damaged area <25%; 2 = renal tubular damaged area 25–50%; 3 = renal tubular damaged area 51–75%; 4 = damaged area of renal tubules >75%.

### Immunofluorescent staining

The kidney tissue samples were dehydrated in 4% paraformaldehyde, and then paraffin-embedded for sectioning. After dewaxing, renal tissue slices were stained with first antibodies and then fluorescently labeled secondary antibody. The tissue slices were photographed with an upright microscope, and 10 high-power fields (200 ×). ImageJ software (National Institutes of Health, Bethesda, MD) was used to measure the fluorescence intensity of the fluorescent stained section.

### Western blot analysis

The kidney tissue samples were homogenized with RIPA Lysis Buffer containing a protease inhibitor cocktail. Proteins (30 μg) were separated by SDS-PAGE and transferred to PVDF membranes. After incubation with 5% nonfat milk for 1 h at room temperature, membranes were incubated with a primary antibody overnight at 4°C and then with fluorescent-conjugated secondary antibody for 1 h at room temperature. ImageJ software (National Institutes of Health, Bethesda, MD) was used to analyze the gray value of western blot results.

### Statistical analysis

Data depicted in graphs represent the means ± SEM for each group. Statistical analysis was performed by Graphpad Prism (version 8.4.0). One-way ANOVA test was performed on the data between the experimental groups, Tukey test was performed to compare multiple means, and the LSD-t test was performed on the data between the two groups. Statistically significant difference between mean values was *p* < 0.05.

## Results

### Expression levels of class IIa HDACs in the kidney of folic acid and I/R-induced acute kidney injury in mice

**FIGURE 1:**
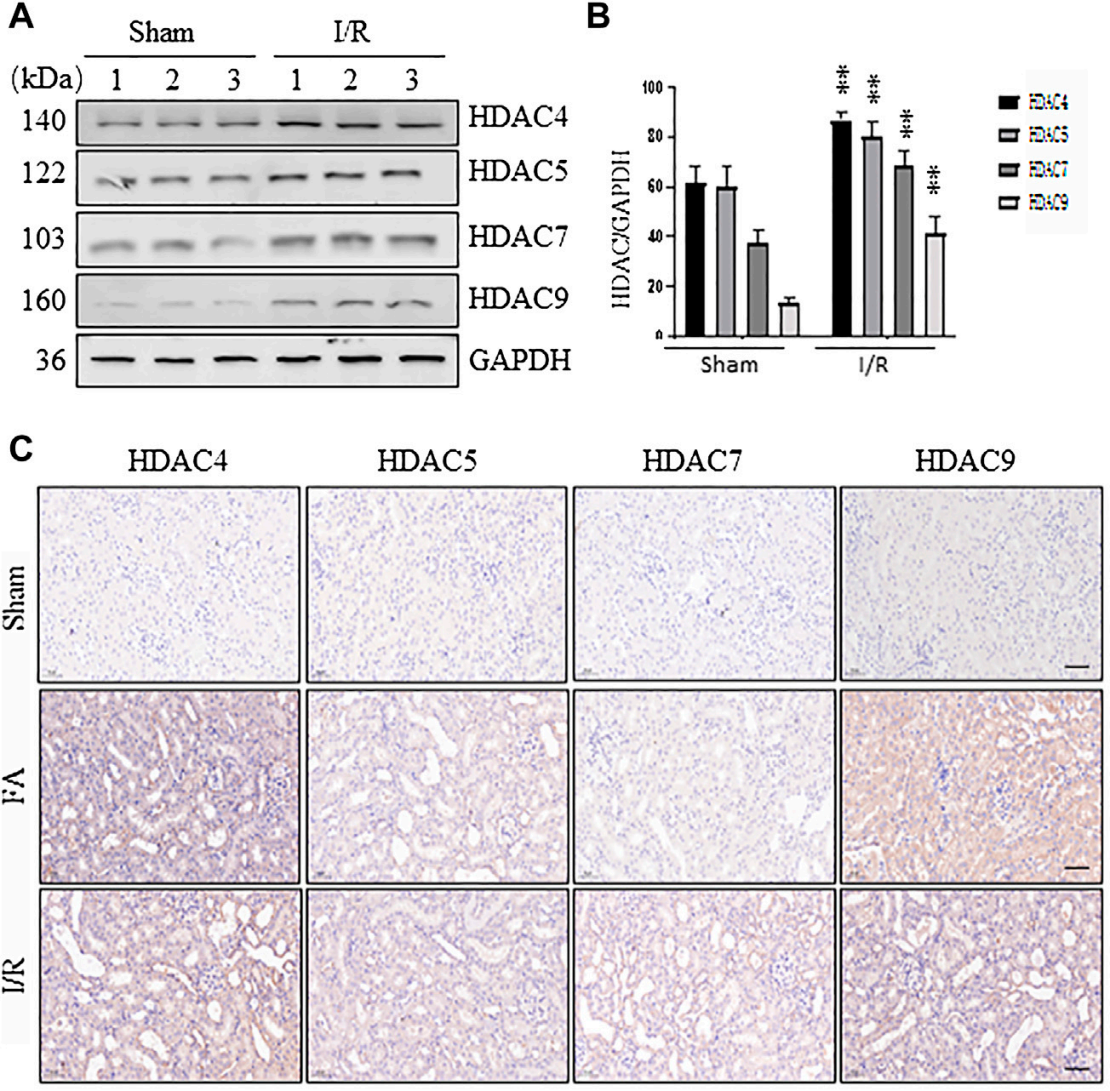
Expression of HDAC4, HDAC5, HDAC7, and HDAC9 in the kidney following ischemia/reperfusion (I/R) and folic acid (FA) injury **(A)** At 48 h after sham operation or I/R, kidneys were collected and tissue lysates were subjected to immunoblot analysis with antibodies against HDAC4, 5, 7, 9, and GAPDH. Representative immunoblots are three samples in each group **(B)** Expression levels of HDAC4, 5, 7, 9 were quantified by densitometry and normalized with GAPDH. Data are expressed as means ± SD (*n* = 3). **p < 0.01 **(C)** At 48 h after sham operation, FA, or I/R, kidneys were collected and tissue sections were subjected to immunohistochemical staining followed by photomicrograph illustrating protein expression of individual class IIa HDACs. Scale bar, 20 µm. Original magnification, ×200.

Our recent studies have shown that all four class IIa HDACs (4, 5, 7, 9) are expressed in the renal tubular cells in a murine model of chronic injury induced by unilateral ureteral obstruction (Zhang et al., 2020). To understand the functional role of class IIa HDACs in AKI, we further examined the expression of HDAC4, HDAC5, HDAC7, and HDAC9 in the kidney of AKI induced by either FA or I/R. Immunoblot analysis showed that all the four class IIa HDACs were expressed in sham-operated kidneys, and their expression levels were significantly increased following either FA or I/R injury, with HDAC4 being more abundant (Figures 1A,B, data not shown). Immunohistochemical staining also demonstrated that either FA or I/R injury to the kidney increased expression of HDAC4, HDAC5, HDAC7, and HDAC9. Notably, all of them were expressed in the cytosol of renal tubular cells (Figure 1C), consistent with what we had observed in UUO injured kidneys (Xiong et al., 2019). These data suggest that class IIa HDACs may play a potential role in regulating renal tubular cell function.

### Administration of TMP269 improves renal function and alleviates pathological changes to kidneys in murine models of folic acid and I/R-induced acute kidney injury

**FIGURE 2:**
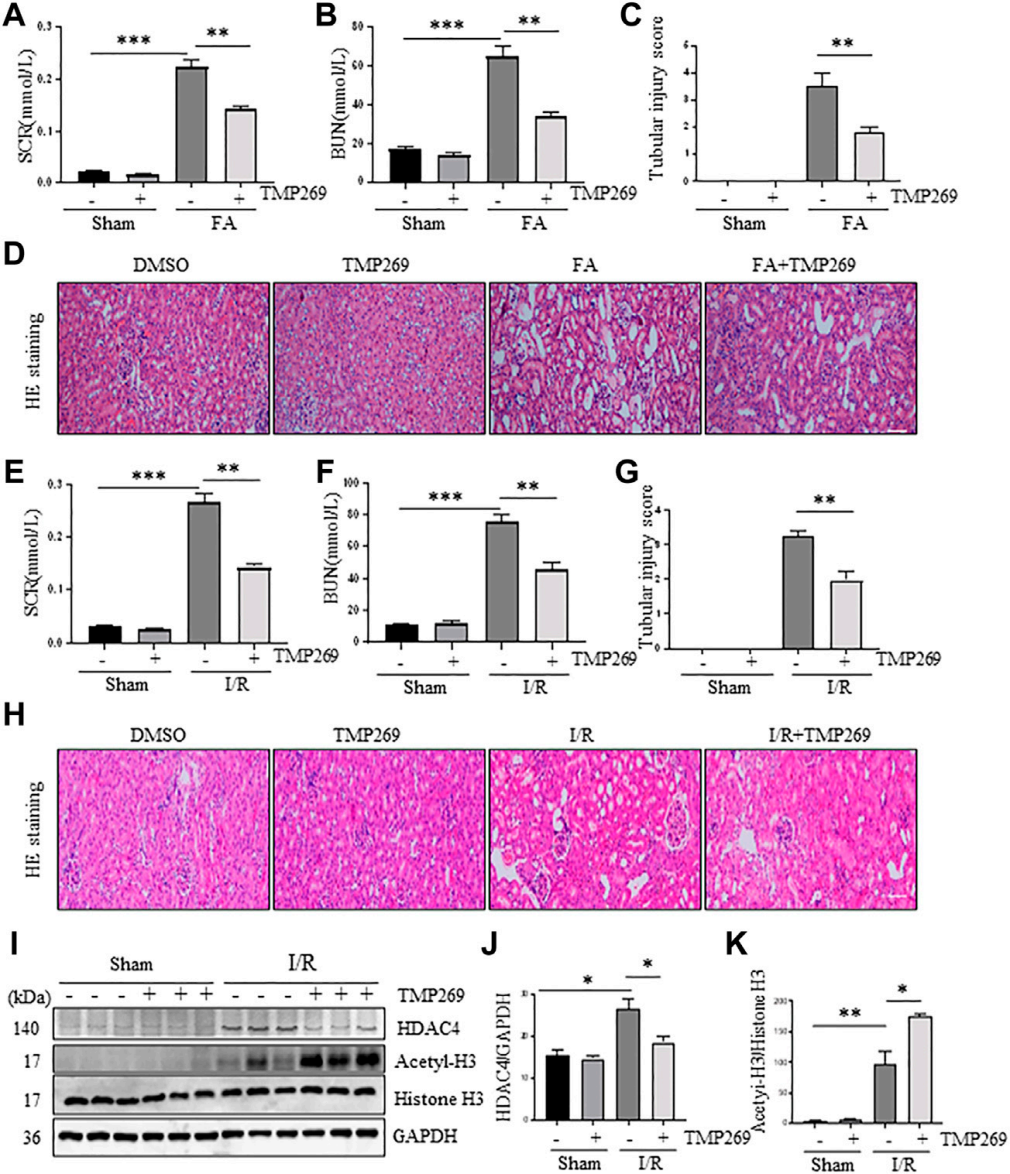
Administration of TMP269 improves renal function and attenuates renal pathological changes in the kidney following FA and I/R injury. At 48 h after various treatments as indicated, blood was collected and the serum creatinine and blood urea nitrogen (BUN) were measured **(A,B,E,F)**. The kidneys injured by FA **(D)** and I/R **(H)** underwent hematoxylin and eosin (HE) staining. Scale bar, 20 µm. Original magnification, ×200. Morphological changes induced by FA **(C)** and I/R **(G)** and were scored based on the scale described in Material and methods **(I)** After IR, kidney tissue lysates were subjected to immunoblot analysis with specific antibodies against HDAC4, acetyl-histone H3 (acetyl-H3), histone H3 and GAPDH. Expression levels of HDAC4 **(J)** and acetyl-histone histone 3 **(K)** were quantified by densitometry and normalized with GAPDH and histone H3, respectively. Data are represented as means ± SD (*n* = 3). **p < 0.01; ***p < 0.001.

To determine the role of class IIa HDACs in AKI, we first examined the effect of TMP269 on the changes in serum creatinine (Scr) and blood urea nitrogen (BUN) levels in murine models of FA or I/R-induced AKI. As shown in Figures 2A,B,E,F, serum creatinine and blood urea nitrogen levels increased following FA-AKI and I/R-AKI. Administration of TMP269 significantly reduced their levels in both models. Next, we examined the effect of TMP269 on renal pathological changes. As shown in Figures 2C,D,G,H, HE staining of kidney tissue sections showed that the control group had no obvious abnormalities; the FA and I/R-AKI injured mice displayed severe damage to renal tubules, as evidenced by tubule dilation, cellular debris accumulated in the tubular lumen, interstitial edema, and inflammatory cell infiltration. Treatment with TMP269 significantly attenuated these pathological changes. The efficacy of TMP269 in inhibiting class IIa HDACs was illustrated by increased histone H3 acetylation injured kidney following ischemia/reperfusion. TMP269 was also able to suppress HDAC4 expression (Figures 2I–K). Similar results were also observed in the kidney of FA-treated mice (data not shown). Taken together, these data suggest that class IIa HDACs play a pathological role in AKI.

### TMP269 reduces renal tubule injury and inhibits renal tubular cell apoptosis in murine models of folic acid and I/R-induced acute kidney injury

**FIGURE 3:**
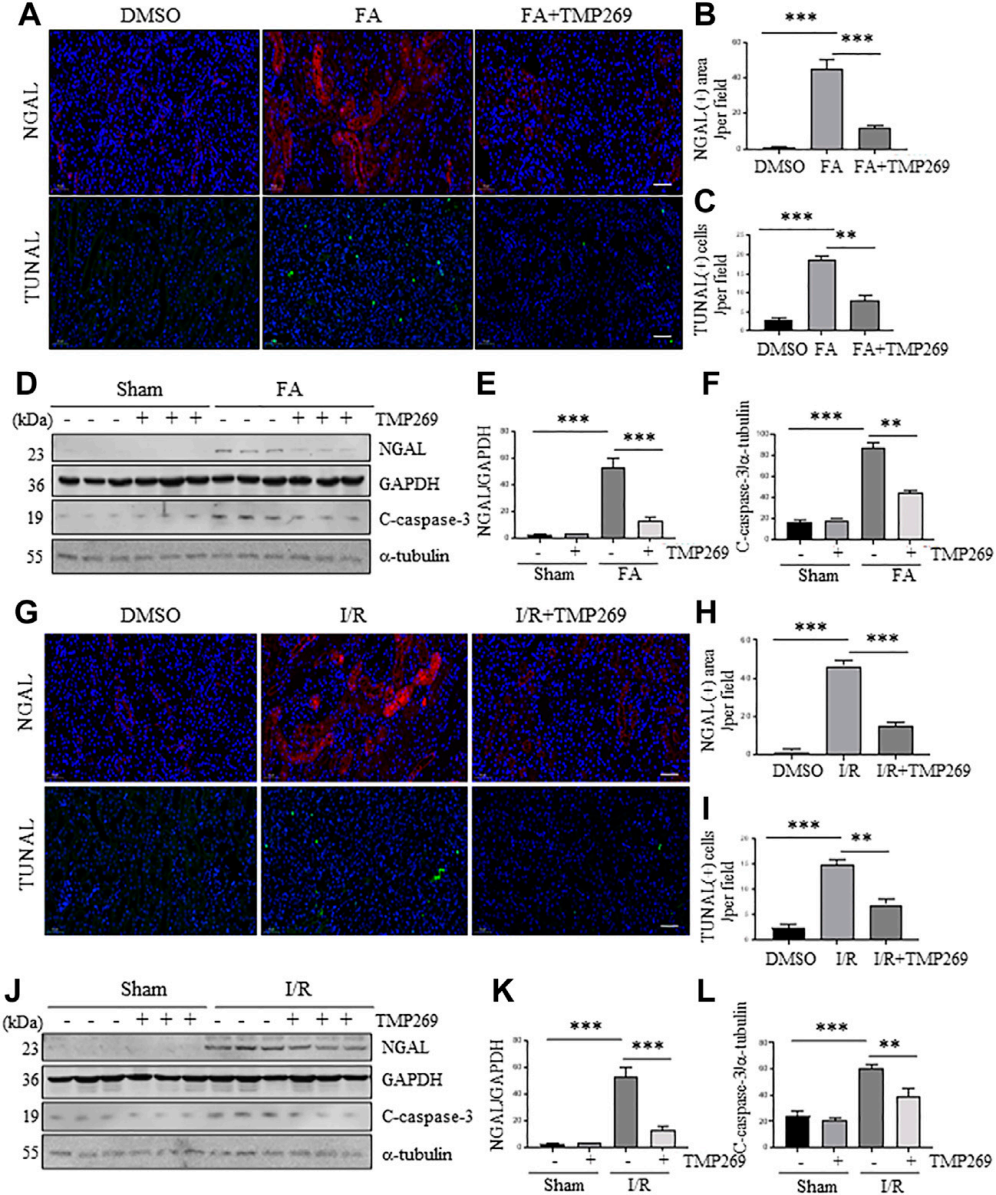
Administration of TMP269 alleviates renal tubular injury and renal tubular cell apoptosis in the kidney following FA and I/R injury. Photomicrographs (×200) illustrate NGAL or TUNAL immunofluorescent staining of the kidney tissues collected at 48 h after FA **(A)** or I/R **(G)** injury with or without TMP269 treatment. Scale bar, 20 µm **(B,H)** Tubules with positive NGAL staining or **(C,I)** tubular cells with TUNAL staining were counted in 10 high-power fields and expressed as means ± SD, respectively. Scale bar, 20 µm. Original magnification, ×200 **(D,J)** Kidneys injured by IR or FA, respectively as indicated, kidney tissue lysates were subjected to immunoblot analysis with specific antibodies against NGAL, GAPDH, cleaved caspase-3 (C-caspase-3) or α-tubulin. Expression levels of NGAL **(E,K)** and cleaved caspase-3 **(F,L)** were quantified by densitometry and normalized with GAPDH and α-tubulin, respectively. Data are represented as means ± SD (*n* = 3). **p < 0.01; ***p < 0.001.

NGAL is expressed in damaged renal tubules and recognized as an AKI biomarker (Shang and Wang, 2017) while renal tubular cell apoptosis is the major pathological change in AKI (Linkermann et al., 2014). We thus examined the effect of TMP269 on the expression of NGAL expression and apoptosis in FA and I/R-induced AKI models by immunofluorescent staining and immunoblot analysis. As shown in Figures 3A,B,G,H, expression of NGAL and TUNEL positive cells (indicative of apoptosis) were observed in some tubules of the kidney subjected to either FA or IR, which were significantly reduced by TMP269 treatment. Western blot analysis also demonstrated that TMP269 treatment inhibited FA or I/R induced expression of NGAL in the kidney (Figures 3D,E, J,K). In addition, TMP269 was effective in suppressing cleavage of caspase 3, a major protease that mediates apoptosis. Notably, a basal level of cleaved caspase-3 was detected in the sham-operated kidney; TMP269 treatment did not significantly affect its expression (Figures 3D,F,J,L). Therefore, activation of class IIa HDACs contributes to apoptosis of renal tubular cells.

### TMP269 regulates the expression and activation of apoptosis-related signal molecules in the kidney following folic acid and I/R-induced acute kidney injury

**FIGURE 4:**
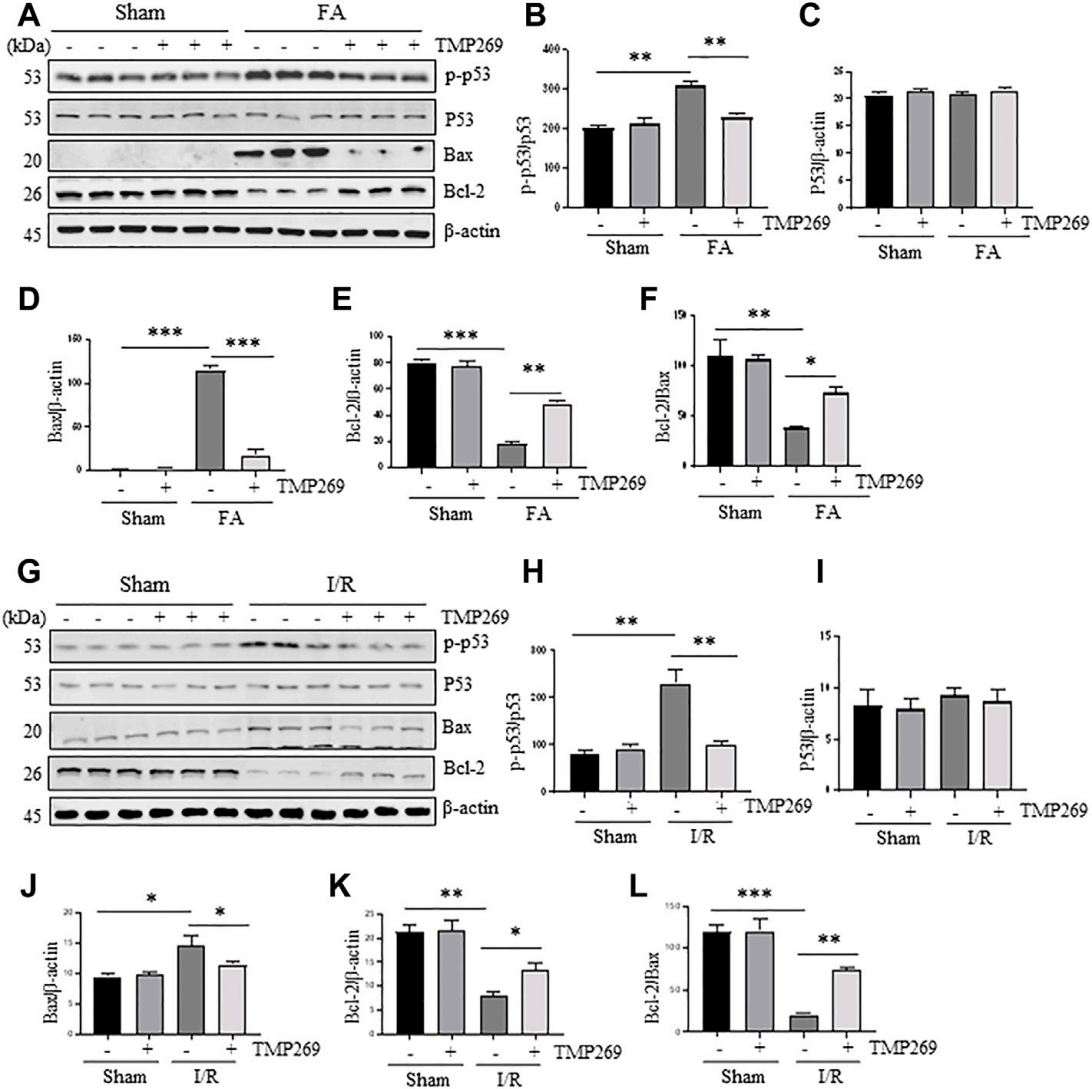
Administration of TMP269 regulates the expression and activation of apoptosis-related signal molecules in the kidney following FA and I/R injury **(A,G)** Kidneys were injured by FA or IR, respectively, and tissue lysates were subjected to immunoblot analysis with specific antibodies against phospho-p53 (p-p53), p53, Bax, Bcl-2 or β-actin. Representative immunoblots are three samples in each group **(B,H)** Expression levels of phospho-p53 (p-p53) were quantified by densitometry and normalized with p53 **(C–E, I–K)** Expression levels of p53, Bax or Bcl-2 were quantified by densitometry, respectively and normalized with b-actin, respectively **(F,L)** The ratio of Bcl-2/Bax were calculated. Data are means ± SD (*n* = 3). *<0.05; **p < 0.01; ***p < 0.001.

It has been reported that renal tubular cell apoptosis in AKI is closely related to the activation of p53, up-regulation of Bax and down-regulation of Bcl-2 (Saikumar and Venkatachalam, 2003). Therefore, we examined the effect of TMP269 on p53 phosphorylation at serine 15 and expression of Bax and Bcl-2. Figures 4A–E shows that expression levels of phosphorylated p53 (p-p53), p53, and Bax were increased in the kidney after FA while Bcl-2 was down-regulated in this model. TMP269 treatment significantly reduced the expression levels of p-p53 and Bax, and partially restored the expression of Bcl-2, whereas expression of total p53 was not affected. Statistical results showed that Bcl-2/Bax, a reactive anti-apoptotic indicator, significantly decreased in AKI, while the ratio recovered significantly in the injured kidney after TMP269 treatment (Figure 4F). Similar results were also observed in the kidney after I/R injury (Figures 4G–L). These results suggest the class IIa HDACs may mediate renal tubular cell apoptosis through mechanisms associated with the activation of p53 and the change in the ratio of the Bcl-2/Bax.

### TMP269 increases autophagy of renal tubular cells in kidneys following folic acid and I/R -induced acute kidney injury

**FIGURE 5:**
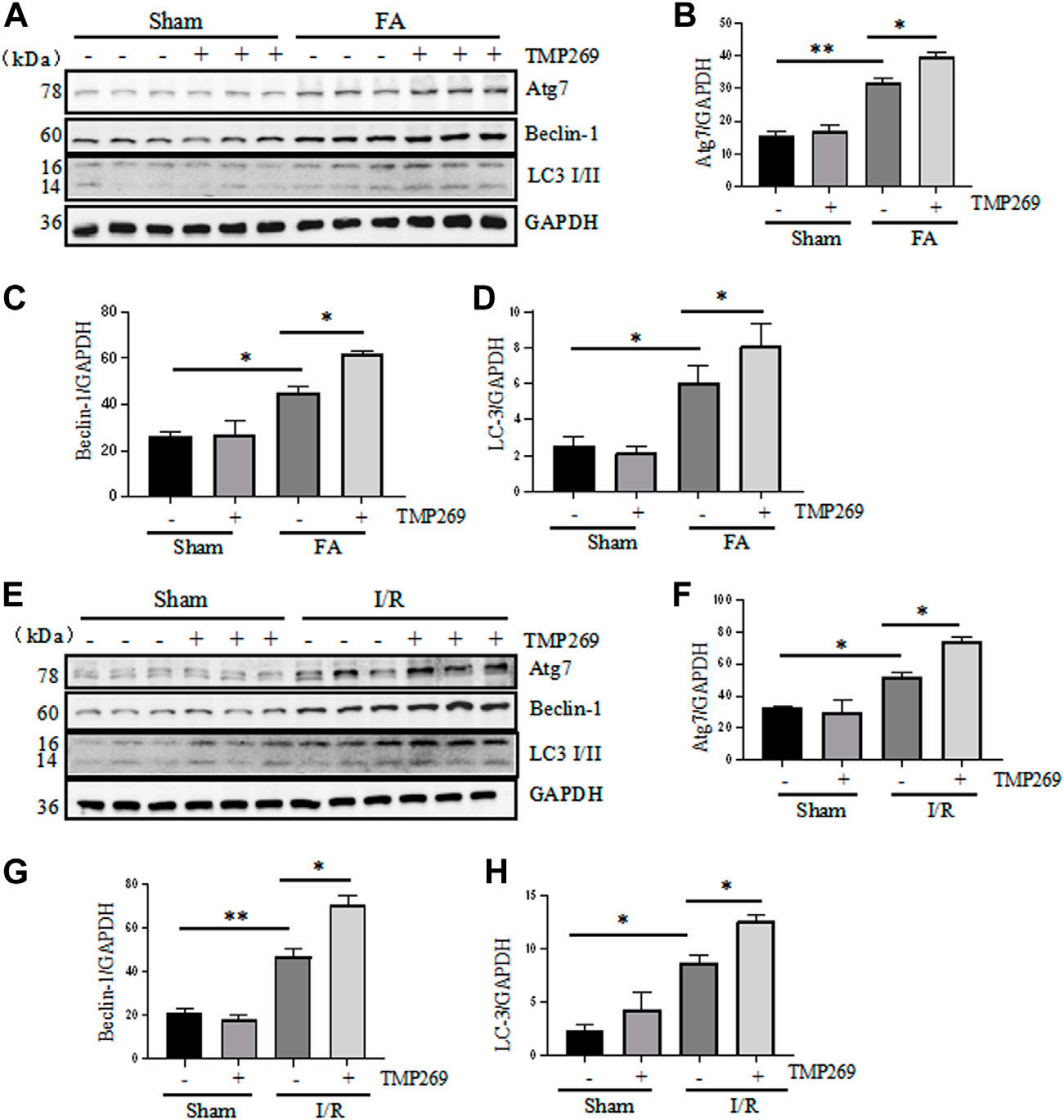
Administration of TMP269 enhances autophagy in the kidney following FA and I/R injury **(A,E)** Kidneys were injured by FA or IR, respectively, and tissue lysates were subjected to immunoblot analysis with specific antibodies against Atg7, Beclin-1, LC3, and GAPDH. Representative immunoblots are three samples in each group. Expression levels of Atg7 **(B,F)**, Beclin-1 **(C,G)**, or LC3 **(D,H)** were quantified by densitometry and normalized with GAPDH. Data are means ± SD (*n* = 3). *<0.05; **p < 0.01; ***p < 0.001.

In addition to apoptosis, injury to the kidney can also induce autophagy in which cells degrade damaged organelles and macromolecules by lysosomes under the regulation of related genes, a process required for cell survival in AKI (Crotzer and Blum, 2010). Therefore, we proceeded to examine the effect of class IIa HDAC inhibition on autophagy in the kidney. The results showed that FA- or I/R-induced AKI resulted in autophagy, as manifested by increased expression levels of three autophagy-related proteins Atg7, beclin-1 and LC3-II. Interestingly, the expression levels of these proteins were further increased in the injured kidney exposed to TMP269 (Figures 5A–H). Since autophagy plays a protective role in AKI induced by FA and I/R (Kaushal and Shah, 2016), these data suggest that TMP269-elicited increase in autophagy may contribute to its renoprotective effects in AKI.

### TMP269 inhibits downregulation of E-cadherin and increases expression of BMP7 and Klotho in the kidney following folic acid and I/R-induced acute kidney injury

**FIGURE 6:**
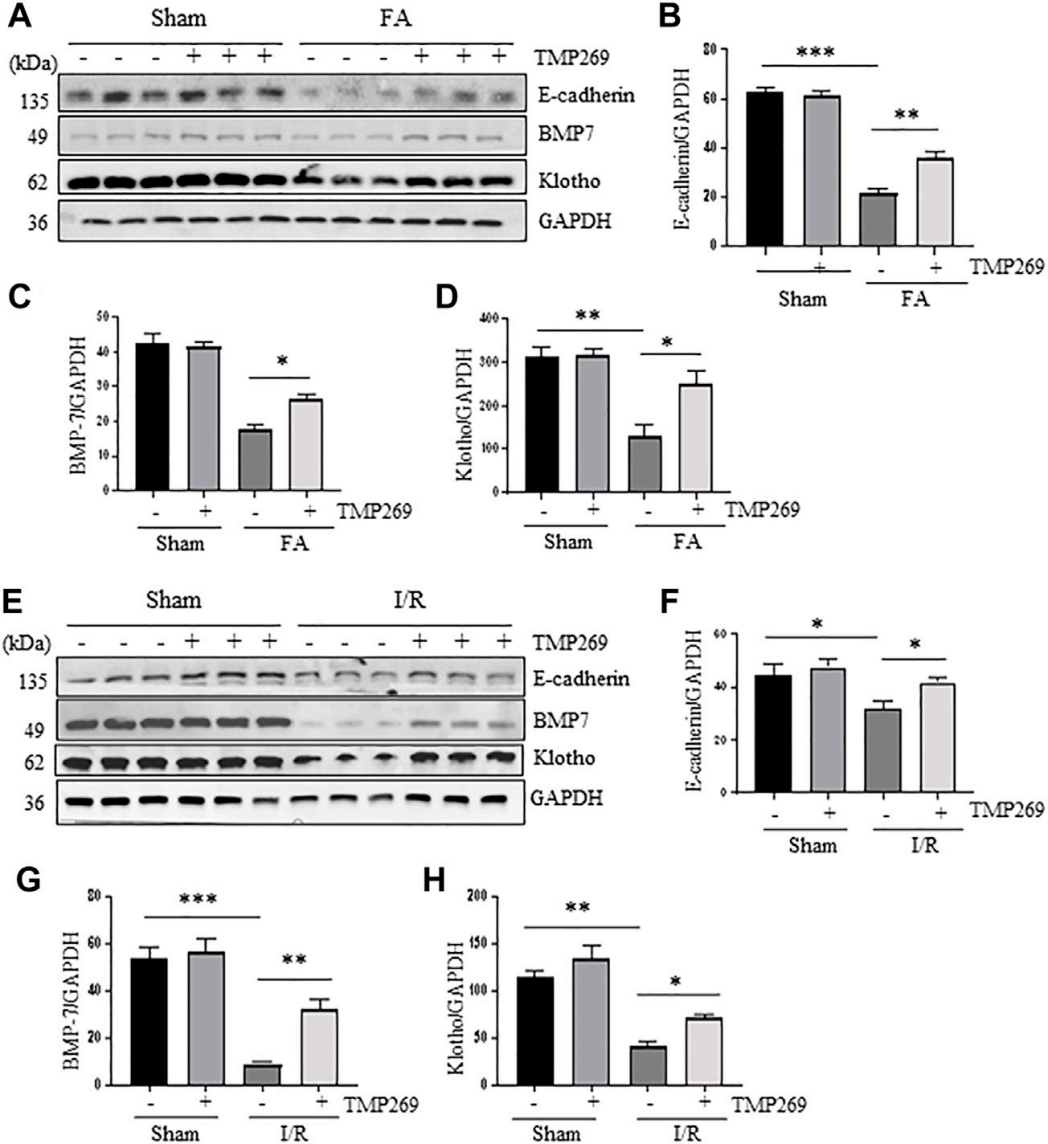
Administration of TMP269 preserves expression of E-cadherin, BMP7 and Klotho in the kidney following FA and I/R injury **(A,E)** Kidneys were injured by FA or IR, respectively, and tissue lysates were subjected to immunoblot analysis with specific antibodies against E-cadherin, BMP7, Klotho and GAPDH. Representative immunoblots are three samples in each group. Expression levels of E-cadherin **(B,F)**, BMP-7 **(C,G)**, or Klotho **(D,H)** were quantified by densitometry and normalized with GAPDH. Data are means ± SD (*n* = 3). *<0.05; **p < 0.01; ***p < 0.001.

E-cadherin is one of the adhesion molecules expressed between epithelial cells and plays an important role in maintaining cell integrity (Rabb, 1994). Injury to the kidney resulted in decreased expression of E-cadherin, which was related to apoptosis and shedding of renal tubular epithelial cells after AKI (Brenner, Weinmann et al., 1997). BMP7, a bone morphogenetic protein (Simon et al., 1999) and Klotho are required for protecting kidneys from acute and chronic injury (Hu and Moe, 2012). As shown in Figures 6A–H, E-cadherin, BMP7 and Klotho were highly expressed in the sham-operated kidneys, and TMP269 treatment did not alter their expression levels. By contrast, FA and I/R significantly reduced renal expression of all those three proteins, whereas TMP269 treatment largely preserve their expression levels under these pathological conditions. This suggests that TMP269-eliciated renal protection in AKI may also be associated with preventing downregulation of E-cadherin, BMP7 and Klotho in the kidney.

### TMP269 promotes cell proliferation in kidneys following folic acid and I/R-induced acute kidney injury

**FIGURE 7:**
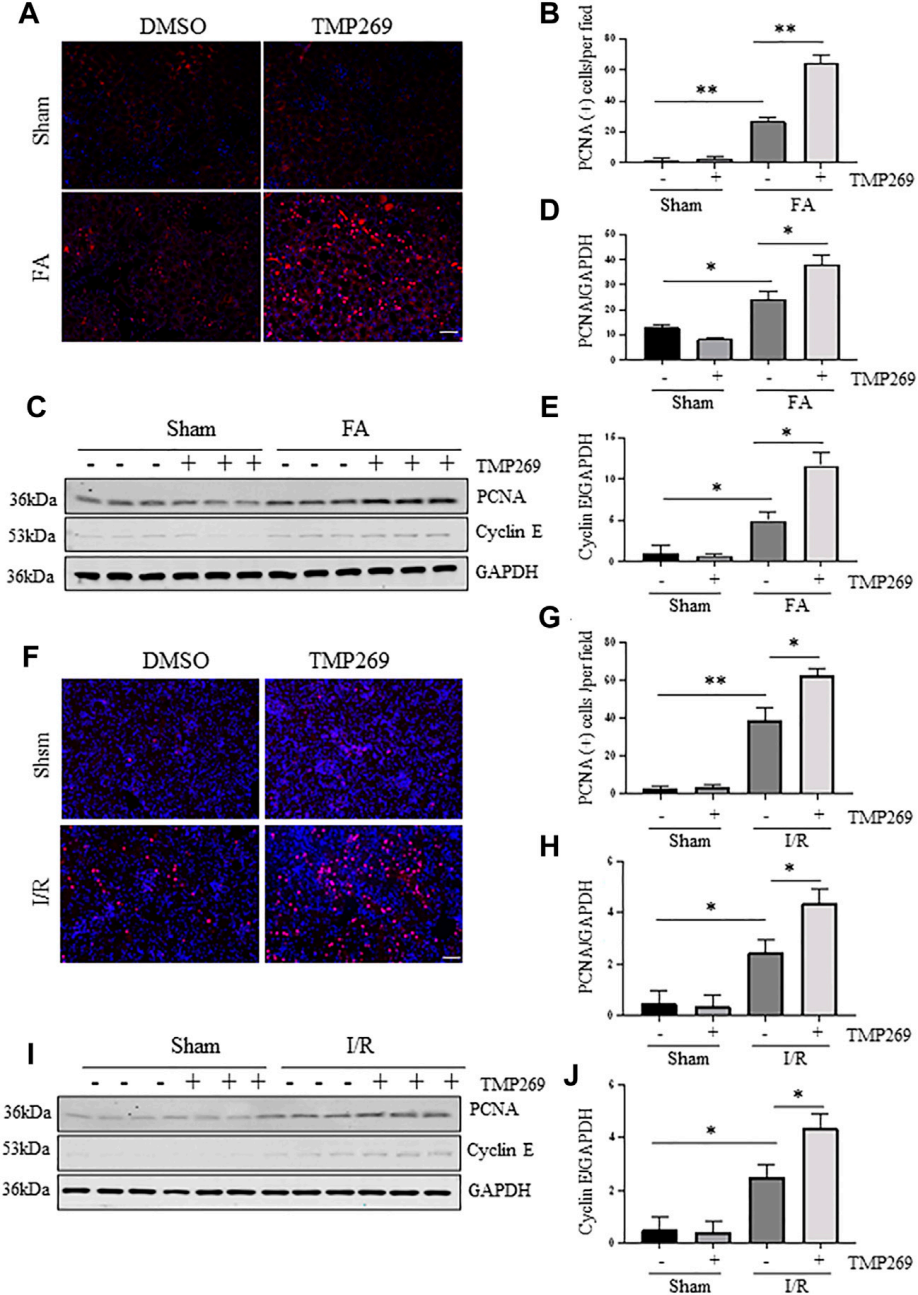
Administration of TMP269 promotes cell proliferation in the kidney following FA and I/R injury **(A,F)** Photomicrographs (×200) illustrate proliferating cell nuclear antigen (PCNA) immunofluorescent staining of the kidney tissues collected from murine models of FA or I/R-induced AKI. Scale bar, 20 µm. Original magnification, ×200 **(B,G)** Tubular cells with positive PCNA staining were counted in 10 high-power fields and expressed as means ± SD **(C,I)** Kidney tissue lysates were subjected to immunoblot analysis with specific antibodies against PCNA, Cyclin E or GAPDH. Representative immunoblots are three samples in each group. Expression levels of PCNA **(D,H)** and Cyclin E **(E,J)** were quantified by densitometry and normalized with GAPDH, respectively. Data are means ± SD (*n* = 3). *<0.05; **p < 0.01; ***p < 0.001.

Renal repair and regeneration are concurrently initiated with renal tubular injury following AKI. During this process, Survival renal tubular cells are first dedifferentiated and then proliferated to replace the lost cells (Ferenbach and Bonventre, 2015). We examined whether blocking class IIa HDACs would also affect renal tubular cell proliferation using proliferating cell nuclear antigen (PCNA) and cyclin E as markers (Duccio et al., 2015). As shown in Figure 7, compared with control kidney, more PCNA (+) tubular cells were observed in the injuried kidney. Interestingly, treatment with TMP269 further increased this population. Immunoblot analysis also indicated that FA and I/R injury increased expression of PCNA and cyclin E; TMP269 treatment enhanced the expression of these proteins. On this basis, we suggest that inhibition of class IIa HDAC can promote the regeneration of renal tubular epithelial cells during AKI.

### TMP269 inhibits activation of MAPK signaling pathways induced by folic acid and I/R during acute kidney injury

**FIGURE 8:**
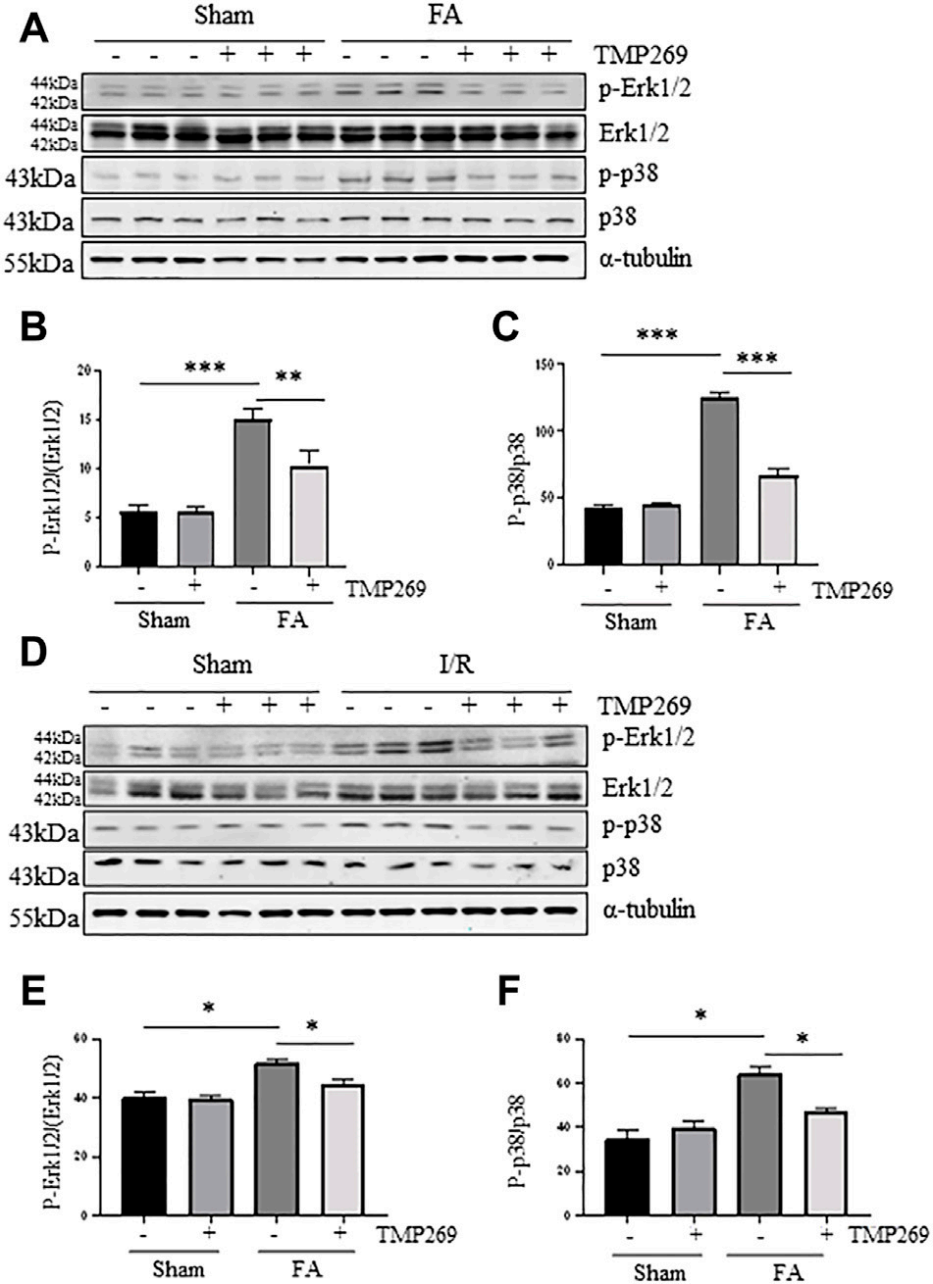
Administration of TMP269 inhibits MAPK signaling pathways **(A,D)** Kidneys were injured by FA or IR, respectively, and tissue lysates were subjected to immunoblot analysis with specific antibodies against p-ERK1/2, ERK1/2, p-p38, p38 or α-tubulin. Representative immunoblots are three samples in each group. Expression levels of p-ERK1/2 **(B,E)** and p-38 **(C,F)** were quantified by densitometry and normalized with ERK1/2 and p38, respectively. Data are means ± SD (*n* = 3). *<0.05; **p < 0.01; ***p < 0.001.

Activation of Erk1/2 and p38 signaling pathways are critically involved in renal tubular cell apoptosis (Ramesh and Reeves, 2005; Zhuang et al., 2007; Liu et al., 2017). We found that either FA or I/R injury increased phosphorylation of ERK1/2 and p38 whereas treatment with TMP269 reduced their phosphorylation levels. Expression of total Erk1/2 and p38 remained unchanged in the kidney after injury (Figures 8A–F). These data suggest that ERK1/2 and p38 pathways may participate in the class IIa HDACs-elicited mechanism leading to AKI.

## Discussion

AKI is induced by many insults, including sepsis, nephrotoxins and I/R. In the current study, we examined the role of class IIa HDACs in murine models of AKI induced by FA and I/R. Our results demonstrated that inhibition of class IIa HDACs with TMP269 reduced levels of serum creatinine and BUN and attenuated renal damage in both models. TMP269 treatment also inhibited renal tubular cell injury and apoptosis, and enhanced autophagy and proliferation. Moreover, TMP269 was effective in suppressing p53 phosphorylation and preventing downregulation of E-cadherin, Klotho and BMP7, three renoprotective proteins. Collectively, these data indicate that class IIa HDACs are critically involved in the pathological processes of AKI and suggest that targeting class IIa HDACs could be a novel strategy for the treatment of AKI.

Compared with class I and III HDACs, class IIa HDACs are less studied in the field of kidney diseases (Verdin et al., 2003). An early report showed that HDAC4 is highly expressed in podocytes of mice with diabetic nephropathy, whereas knocking down this HDAC isoform with siRNA alleviates podocyte damage *in vivo* and *in vitro*, suggesting the importance of HDAC4 in mediating podocyte injury (Wang et al., 2014). Our recent studies showed that all the class IIa HDAC isoforms are expressed in the kidney, with HDAC4 being more abundant in renal tubular cells after unilateral ureteral obstruction; blocking class IIa HDACs with MC1568 exhibited an anti-fibrotic effect (Xiong et al., 2019). In another study, we found that blocking class IIa HDACs with TMP195 protects against LPS-induced renal tubular damage (Zhang et al., 2020). In agreement with those observations, FA and I/R also increased the expression of class IIa HDACs, in particular HDAC4, in the kidney, while inhibition of class IIa HDACs with TMP269 reduced the renal damage and improved renal function. Given that HDAC4 is abundantly induced by both acute and chronic injuries, it is likely that HDAC4 is the major isoform that mediates various renal pathogenetic processes, leading to acute and chronic kidney injury. Further studies using conditional knockout of HADC4 will clarify its role in these processes.

Our understanding of the mechanism by which inhibition of class IIa HDACs confers renoprotection in AKI remains incomplete. Here we show that administration of TMP269 dramatically inhibited renal tubular cell apoptosis in these two models of AKI, as indicated by reduced number of TUNEL-positive cells and decreased expression of Bax and cleaved caspase-3 while preserved Bcl2 expression. In addition, TMP269 was also effective by enhancing renal tubular cell autophagy. Although induction of autophagy plays either a detrimental or a beneficial role, depending on cell type and insults, it has been generally recognized that basal autophagy in the kidney is vital for the normal homeostasis of the proximal tubules and increased autophagy has a renoprotective effect in animal models of AKI (Periyasamy-Thandavan et al., 2008; Jiang et al., 2012). On this basis, TMP269-elicited renoprotective effects may occur through at least two mechanisms, inhibiting renal epithelial cell apoptosis and enhancing autophagy. The detailed molecular mechanism of class IIa HDACs-mediated these processes may be multifaceted, we demonstrated that TMP269 treatment inhibited the phosphorylation of p53, a well-known tumor suppressor that contributes to AKI by regulating apoptosis and autophagy (Yan et al., 2016; Tang et al., 2019), and preserves expression of E-cadherin, Klotho and BMP-7, three proteins that protect against AKI (Hu and Moe, 2012; Manson et al., 2015; Ma et al., 2017; Gao et al., 2018). In addition, since activation of ERK1/2 and p38 is also involved in renal tubular cell apoptosis (Ramesh and Reeves, 2005; Zhuang et al., 2007; Liu et al., 2017), and our data showed that TMP269 treatment reduced their phosphorylation, it is possible that class IIa HDACs also contribute to renal tubular apoptosis through activation of these two pathways. Additional studies are needed to elucidate the mechanism by which class IIa HDACs induce ERK1/2 and p38 activation.

E-cadherin-mediated formation of adhesive complexes is critical for stabilizing the surfaces of neighboring cells and survival. Class IIa HDACs inhibition may also exhibit renal protective response though promoting preservation of E-cadherin expression. Normal tubular epithelial cells are connected to each other to maintain their structural and functional integrity. During AKI, expression of E-cadherin is reduced and the E-cadherin/catenin junction complex is destroyed in the kidney, resulting in the disruption of cell–cell adhesion and shedding of renal tubular epithelial cells (Brenner et al., 1997; Ueno et al., 2015). In this study, we observed that E-cadherin expression in FA-induced AKI was significantly reduced, but largely maintained in AKI kidneys treated with TMP269, suggesting that inactivation of class IIa HDACs resulted in restoration of E-cadherin expression in the injured kidney. Currently, the pharmacological mechanism of class IIa HDACs inhibition elicited preservation of E-cadherin expression remains undefined in the kidney. A recent study showed that E-cadherin can be acetylated at lysine^870^ and at lysine^871^ by CREB-binding protein in tumor cells (Zhao et al., 2019). This acetylation is associated with the released β-catenin from the E-cadherin/β-catenin complex, leading to loss of membranous E-cadherin and increased nuclear localization of E-cadherin. As such, it would be interesting to examine whether E-cadherin acetylation also occurs in the injured kidney and is subject to the regulation by class IIa HDACs.

Klotho and BMP7 play an important role in protecting renal tubular cells against apoptosis (Simon et al., 1999; Gould et al., 2002; Archdeacon and Detwiler, 2008). Emerging evidence indicates a regulatory role for HDACs in Klotho and BMP7 expression. Marumo et al., observed that treatment with class I/II HDACs inhibitor trichostatin A can induce the expression of BMP7 mRNA in cultured renal tubular cells (Yoshikawa et al., 2007) and in I/R injured kidneys (Marumo et al., 2008). Lin et al., also demonstrated that trichostatin A restores Klotho expression through activation of peroxisome proliferation-activated receptor-γ in the kidneys of adenine-fed CKD mice (Lin et al., 2017a). Our results showed that renal expression of both Klotho and BMP7 was reduced in mice with AKI, but that TMP269 treatment restored their expression. Consistent with this observation, selective inhibition of class IIa HDACs with another inhibitor TMP195 also led to preservation of renal BMP7 in a murine model of AKI induced by lipopolysaccharide (Zhang et al., 2020). Moreover, blocking class IIa HDACs with MC1568 reversed UUO injury-induced downregulation of Klotho and BMP7 in the fibrotic kidney (Xiong et al., 2019). These data suggest that Klotho and BMP7 are key genes involved in the HDAC aberration-associated acute and chronic injuries, and that restoration of endogenous Klotho and BMP7 is a critical molecular event that contributes to renoprotection by HDAC inhibition. Mechanistic studies show that HDAC inhibition can increase BMP7 and Klotho expression by reversing the promoter hypoacetylation and transcriptional activation (Lin et al., 2017b; Kong et al., 2021), suggesting that both BMP7and Klotho promoters are subjected to epigenetic regulation, in particular, acetylation.

Class IIa HDAC inhibition may also contribute to renoprotection by promoting renal regeneration. It is well known that proliferation of remaining renal tubular epithelial cells after AKI is a key step in the recovery of damaged epithelium (Duccio et al., 2015). Since PCNA is a nucleoprotein expressed in actively proliferating cells, and its increased expression is a sign of increased cell proliferation, we examined the effect of class IIa HDACs inhibition on PCNA expression. We found that treatment with TMP269 increased the number of PCNA (+) tubular cells and promoted PCNA expression in the kidney tissue after injury, suggesting that class IIa HDAC inhibition promotes renal tubular cell proliferation during AKI. Currently, it remains unclear about how class IIa HDACs regulate the expression of PCNA. It has been reported that treatment with TSA, a pan HDACI/II inhibitor, can induce PCNA acetylation and acetylated PCNA is involved in DNA replication, whereas deacetylation of PCNA is associated with the termination of DNA replication (Naryzhny and Lee, 2004). Therefore, class IIa HDAC inhibition may also promote renal regeneration and functional recovery through inducing PCNA acetylation directly.

Class IIa HDAC inhibitors have been extensively tested in animal models of various diseases, however, an HDAC-based approach or agent has not been used for the treatment of kidney diseases. Nevertheless, four pan-HDAC inhibitors, vorinostat, romidepsin, belinostat, and panobinostat, have been approved by the US FDA for treating cutaneous T cell lymphoma, peripheral T cell lymphoma, and multiple myeloma (Cappellacci et al., 2020); many clinical trials using other HDAC inhibitors are also underway to treat various diseases, especially cancers (Cappellacci et al., 2020). Our current study provides evidence that administration of a TMP 269, small-molecule inhibitor of class IIa HDAC, can effectively attenuate renal tubular damage and improve renal function in two preclinical models of AKI. As such, clinical trials of HDAC inhibitors based on the results from preclinical models of kidney diseases may be a promising strategy to develop novel therapeutic treatment for AKI and other kidney diseases.

In summary, we demonstrated that selective inhibition of class IIa HDACs with TMP269 attenuated acute kidney damage and improved renal function in murine models of AKI. The renoprotective effect is associated with inhibition of renal tubular cell apoptosis, enhancing autophage, and cell proliferation. Given that class IIa HDACs mediate both AKI and renal fibrosis, targeting this class of HDACs may be a potential therapeutic treatment for AKI and CKD.

## Data Availability

The original contributions presented in the study are included in the article/Supplementary Material, further inquiries can be directed to the corresponding author.

## References

[B1] Agarwal A. Dong Z. Harris R. Murray P. Parikh S. M. Rosner M. H. X. W. G. A cute Dialysis Quality Initiative (2016). Cellular and molecular mechanisms of AKI. J. Am. Soc. Nephrol. 27 (5), 1288–1299. 10.1681/asn.2015070740 26860342 PMC4849836

[B2] Archdeacon P. Detwiler R. K. (2008). Bone morphogenetic protein 7 (BMP7): A critical role in kidney development and a putative modulator of kidney injury. Adv. Chronic Kidney Dis. 15 (3), 314–320. 10.1053/j.ackd.2008.04.011 18565482

[B3] Brenner B. Weinmann S. Grassmé H. Lang F. Gulbins O. Gulbins E. (1997). L-selectin activates JNK via src-like tyrosine kinases and the small G-protein Rac. Immunology 92 (2), 214–219. 10.1046/j.1365-2567.1997.00336.x 9415029 PMC1364061

[B4] Cappellacci L. Perinelli D. R. Maggi F. Petrelli M. Petrelli R. (2020). Recent progress in histone deacetylase inhibitors as anticancer agents. Curr. Med. Chem. 27 (15), 2449–2493. 10.2174/0929867325666181016163110 30332940

[B5] Costalonga E. C. Silva F. M. Noronha I. L. (2016). Valproic acid prevents renal dysfunction and inflammation in the ischemia-reperfusion injury model. Biomed. Res. Int. 2016, 5985903. 10.1155/2016/5985903 27195290 PMC4852329

[B6] Crotzer V. L. Blum J. S. (2010). Autophagy and adaptive immunity. immunology 131 (1), 9–17. 10.1111/j.1365-2567.2010.03321.x 20586810 PMC2966753

[B7] Duccio L. Francesca B. Paola R. (2015). How much can the tubule regenerate and who does it? An open question. Nephrol. Dial. Transplant. 31, 1243. 10.1093/ndt/gfv262 26175143 PMC4967725

[B8] Falkenberg K. J. Johnstone R. W. (2014). Histone deacetylases and their inhibitors in cancer, neurological diseases and immune disorders. Nat. Rev. Drug Discov. 13 (9), 673–691. 10.1038/nrd4360 25131830

[B9] Ferenbach D. A. Bonventre J. V. (2015). Mechanisms of maladaptive repair after AKI leading to accelerated kidney ageing and CKD. Nat. Rev. Nephrol. 11 (5), 264–276. 10.1038/nrneph.2015.3 25643664 PMC4412815

[B10] Gao L. Liu M. M. Zang H. M. Ma Q. Y. Yang Q. Jiang L. (2018). Restoration of E-cadherin by PPBICA protects against cisplatin-induced acute kidney injury by attenuating inflammation and programmed cell death. Lab. Invest. 98 (7), 911–923. 10.1038/s41374-018-0052-5 29581579

[B11] Gould S. E. Day M. Dorai S. S. Dorai H. Gould S. E. Day M. (2002). BMP-7 regulates chemokine, cytokine, and hemodynamic gene expression in proximal tubule cells. Kidney Int. 61 (1), 51–60. 10.1046/j.1523-1755.2002.00103.x 11786084

[B12] Hu M. C. Moe O. W. (2012). Klotho as a potential biomarker and therapy for acute kidney injury. Nat. Rev. Nephrol. 8 (7), 423–429. 10.1038/nrneph.2012.92 22664739 PMC3752896

[B13] Hyndman K. A. (2020). Histone deacetylases in kidney physiology and acute kidney injury. Semin. Nephrol. 40 (2), 138–147. 10.1016/j.semnephrol.2020.01.005 32303277 PMC7172006

[B14] Jiang M. Wei Q. Dong G. Komatsu M. Dong Y. Dong Z. (2012). Autophagy in proximal tubules protects against acute kidney injury. Kidney Int. 82 (12), 1271–1283. 10.1038/ki.2012.261 22854643 PMC3491167

[B15] Kaushal G. P. Shah S. V. (2016). Autophagy in acute kidney injury. Kidney Int. 89 (4), 779–791. 10.1016/j.kint.2015.11.021 26924060 PMC4801755

[B16] Kellum J. A. Romagnani P. Ashuntantang G. Ronco C. Anders A. Anders H. J. (2021). Acute kidney injury. Nat. Rev. Dis. Prim. 7 (1), 52. 10.1038/s41572-021-00284-z 34267223

[B17] Kong L. Wang H. Li C. Cheng H. Cui Y. Liu L. (2021). Sulforaphane ameliorates diabetes-induced renal fibrosis through epigenetic up-regulation of BMP-7. Diabetes Metab. J. 45, 909. 10.4093/dmj.2020.0168 34082508 PMC8640156

[B18] Lin W. Li Y. Chen F. Yin S. Cao Z. Cao W. (2017a). Klotho preservation via histone deacetylase inhibition attenuates chronic kidney disease-associated bone injury in mice. Sci. Rep. 7, 46195. 10.1038/srep46195 28387374 PMC5384196

[B19] Lin W. Zhang Q. Liu L. Yin S. Cao Z. Cao W. (2017b). Klotho restoration via acetylation of Peroxisome Proliferation-Activated Receptor γ reduces the progression of chronic kidney disease. Kidney Int. 92 (3), 669–679. 10.1016/j.kint.2017.02.023 28416226

[B20] Linkermann A. Chen G. Dong G. Kunzendorf U. Dong S. Dong Z. (2014). Regulated cell death in AKI. J. Am. Soc. Nephrol. 25 (12), 2689–2701. 10.1681/ASN.2014030262 24925726 PMC4243360

[B21] Liu H. (2021). The roles of histone deacetylases in kidney development and disease. Clin. Exp. Nephrol. 25 (3), 215–223. 10.1007/s10157-020-01995-5 33398599 PMC7925501

[B22] Liu J. Chen M. Chen L. (2017). Novel pathogenesis: Regulation of apoptosis by apelin/APJ system. Acta Biochim. Biophys. Sin. (Shanghai) 49 (6), 471–478. 10.1093/abbs/gmx035 28407045

[B23] Liu J. Livingston M. J. Dong G. Tang C. Su Y. Wu G. (2018). Histone deacetylase inhibitors protect against cisplatin-induced acute kidney injury by activating autophagy in proximal tubular cells. Cell Death Dis. 9 (3), 322. 10.1038/s41419-018-0374-7 29476062 PMC5833747

[B24] Liu N. Zhuang S. (2015). Treatment of chronic kidney diseases with histone deacetylase inhibitors. Front. Physiol. 6, 121. 10.3389/fphys.2015.00121 25972812 PMC4411966

[B25] Ma T. Huang C. Xu Q. Yang Y. Liu Y. Meng X. (2017). Suppression of BMP-7 by histone deacetylase 2 promoted apoptosis of renal tubular epithelial cells in acute kidney injury. Cell Death Dis. 8 (10), e3139. 10.1038/cddis.2017.552 29072686 PMC5680919

[B26] Manson S. R. Austin P. F. Moore Q. Moore K. H. (2015). BMP-7 signaling and its critical roles in kidney development, the responses to renal injury, and chronic kidney disease. Vitam. Horm. 99, 91–144. 10.1016/bs.vh.2015.05.003 26279374

[B27] Marumo T. Hishikawa K. Fujita M. Fujita T. (2008). Epigenetic regulation of BMP7 in the regenerative response to ischemia. J. Am. Soc. Nephrol. 19 (7), 1311–1320. 10.1681/ASN.2007091040 18322163 PMC2440290

[B28] Naryzhny S. N. Lee H. (2004). The post-translational modifications of proliferating cell nuclear antigen: Acetylation, not phosphorylation, plays an important role in the regulation of its function. J. Biol. Chem. 279 (19), 20194–20199. 10.1074/jbc.M312850200 14988403

[B29] Periyasamy-Thandavan S. Jiang M. Wei Q. Smith R. Dong X. M. Dong Z. (2008). Autophagy is cytoprotective during cisplatin injury of renal proximal tubular cells. Kidney Int. 74 (5), 631–640. 10.1038/ki.2008.214 18509315

[B30] Rabb H. A. (1994). Cell adhesion molecules and the kidney. Am. J. Kidney Dis. 23 (2), 155–166. 10.1016/s0272-6386(12)80965-6 8311068

[B31] Ramesh G. Reeves W. B. (2005). p38 MAP kinase inhibition ameliorates cisplatin nephrotoxicity in mice. Am. J. Physiol. Ren. Physiol. 289 (1), F166–F174. 10.1152/ajprenal.00401.2004 15701814

[B32] Ruijter A. J. M. D. Gennip A. H. V. Caron H. N. Kemp S. Kuilenburg A. B. P. V. (2003). Histone deacetylases (HDACs): Characterization of the classical HDAC family. Biochem. J. 370 (Pt 3), 737–749. 10.1042/bj20021321 12429021 PMC1223209

[B33] Saikumar P. Venkatachalam M. A. (2003). Role of apoptosis in hypoxic/ischemic damage in the kidney. Semin. Nephrol. 23 (6), 511–521. 10.1053/s0270-9295(03)00130-x 14631559

[B34] Shang W. Wang Z. (2017). The update of NGAL in acute kidney injury. Curr. Protein Pept. Sci. 18 (12), 1211–1217. 10.2174/1389203717666160909125004 27634444

[B35] Shi Y. Xu L. Tang J. Fang L. Ma S. Ma X. (2017). Inhibition of HDAC6 protects against rhabdomyolysis-induced acute kidney injury. Am. J. Physiol. Ren. Physiol. 312 (3), F502–F515. 10.1152/ajprenal.00546.2016 PMC5374306 28052874

[B36] Simon M. Maresh J. G. Harris S. E. Hernandez J. D. Arar M. Abboud M. S. (1999). Expression of bone morphogenetic protein-7 mRNA in normal and ischemic adult rat kidney. Am. J. Physiol. 276 (3 Pt 2), F382–F389. 10.1152/ajprenal.1999.276.3.F382 10070161

[B37] Tang J. Shi Y. Liu N. Xu L. Zang X. Li P. (2018). Blockade of histone deacetylase 6 protects against cisplatin-induced acute kidney injury. Clin. Sci. (Lond) 132 (3), 339–359. 10.1042/CS20171417 29358506

[B38] Tang C. Ma Z. Zhu J. Liu Z. Liu Y. Liu Y. (2019). P53 in kidney injury and repair: Mechanism and therapeutic potentials. Pharmacol. Ther. 195, 5–12. 10.1016/j.pharmthera.2018.10.013 30347214

[B39] Ueno T. Fujisawa M. Nishitani K. Nakai H. Fukuda K. Sasaki Y. (2015). Stimulation of P-selectin glycoprotein ligand-1 on mouse neutrophils activates β 2 -integrin mediated cell attachment to ICAM-1. Eur. J. Immunol. 28 (2), 433–443. 10.1002/(SICI)1521-4141(199802)28:02<433::AID-IMMU433>3.0.CO;2-U 9521050

[B40] Venkatachalam M. A. Weinberg J. M. Bidani W. Bidani A. K. (2015). Failed tubule recovery, AKI-CKD transition, and kidney disease progression. J. Am. Soc. Nephrol. 26 (8), 1765–1776. 10.1681/ASN.2015010006 25810494 PMC4520181

[B41] Verdin E. Kasler F. Kasler H. G. (2003). Class II histone deacetylases: Versatile regulators. Trends Genet. 19 (5), 286–293. 10.1016/S0168-9525(03)00073-8 12711221

[B42] Wang X. Liu J. Zhen J. Zhang C. Wan Q. Liu G. (2014). Histone deacetylase 4 selectively contributes to podocyte injury in diabetic nephropathy. Kidney Int. 86 (4), 712–725. 10.1038/ki.2014.111 24717296

[B43] Xiong C. Guan Y. Zhou X. Liu L. Zhuang M. A. Zhang W. (2019). Selective inhibition of class IIa histone deacetylases alleviates renal fibrosis. FASEB J. 33 (7), 8249–8262. 10.1096/fj.201801067RR 30951378 PMC6593874

[B44] Yan M. Tang C. Ma Z. Dong S. Dong Z. (2016). DNA damage response in nephrotoxic and ischemic kidney injury. Toxicol. Appl. Pharmacol. 313, 104–108. 10.1016/j.taap.2016.10.022 27984128 PMC5362306

[B45] Yoshikawa M. Hishikawa K. Fujita T. Fujita T. (2007). Inhibition of histone deacetylase activity suppresses epithelial-to-mesenchymal transition induced by TGF-beta1 in human renal epithelial cells. J. Am. Soc. Nephrol. 18 (1), 58–65. 10.1681/ASN.2005111187 17135397

[B46] Zhang W. Guan Y. Zhuang G. Zhuang S. (2020). Class IIa HDAC inhibitor TMP195 alleviates lipopolysaccharide-induced acute kidney injury. Am. J. Physiol. Ren. Physiol. 319 (6), F1015–F1026. 10.1152/ajprenal.00405.2020 PMC7792695 33017186

[B47] Zhao S. Li X. Li H. (2018). Beyond histone acetylation-writing and erasing histone acylations. Curr. Opin. Struct. Biol. 53, 169–177. 10.1016/j.sbi.2018.10.001 30391813

[B48] Zhao Y. Yu T. Zhang N. Chen J. Zhang P. Li S. (2019). Nuclear E-cadherin acetylation promotes colorectal tumorigenesis via enhancing β-catenin activity. Mol. Cancer Res. 17 (2), 655–665. 10.1158/1541-7786.MCR-18-0637 30401720

[B49] Zhou X. Chen H. Shi Y. Ma X. Liu S. Liu N. (2021). The role and mechanism of histone deacetylases in acute kidney injury. Front. Pharmacol. 12, 695237. 10.3389/fphar.2021.695237 34220520 PMC8242167

[B50] Zhuang S. Yan Y. Daubert R. A. Schnellmann J. Schnellmann R. G. (2007). ERK promotes hydrogen peroxide-induced apoptosis through caspase-3 activation and inhibition of Akt in renal epithelial cells. Am. J. Physiol. Ren. Physiol. 292 (1), F440–F447. 10.1152/ajprenal.00170.2006 16885155

